# Case report: Shingles-associated probable Bickerstaff brainstem encephalitis with IgM anti-sulfatide positivity

**DOI:** 10.3389/fimmu.2024.1358886

**Published:** 2024-04-09

**Authors:** Xiaoxue Fu, Qianli Zhan, Linjie Zhang, Xiaoyan Tian

**Affiliations:** Baoding First Central Hospital, Baoding, Hebei, China

**Keywords:** Bickerstaff brainstem encephalitis, brainstem encephalitis, herpes zoster, anti-sulfatide antibodies, secondary autoimmune response

## Abstract

**Background:**

Bickerstaff brainstem encephalitis (BBE) is a rare disease considered caused by acute demyelination of the brainstem, most often resulting from secondary autoimmune responses. To our knowledge, this is the first probable case report of shingles-associated BBE with anti-sulfatide IgM positivity.

**Case presentation:**

We report the case of an 83-year-old woman with symptoms of progressive limb weakness, difficulty swallowing food, and disturbed consciousness that occurred 4 weeks following herpes zoster infection. Autoimmune anti-sulfatide antibodies were positive and fluid-attenuated inversion recovery (FLAIR) sequences revealed clear high signal intensity in pons and bilateral thalamus. Our patient’s condition improved markedly with glucocorticoid treatment. After 2 months of treatment, our patient was fully recovered. We considered that for her case, BBE is the most appropriate diagnosis.

**Conclusions:**

We emphasize the importance of a careful medical history and assessment of clinical symptoms, performing MRI, testing autoimmune antibodies for rapid diagnosis, and ruling out differential diagnoses. Further studies involving more patients with BBE with IgM anti-sulfatide autoantibodies will increase the understanding of the clinical characteristics and advance the diagnosis and treatment of this syndrome. Meanwhile, it is crucial for dermatologists to know about this severe neurological complication following shingles.

## Introduction

Shingles is a common infectious viral disorder, with a high incidence in the elderly. Although the eruption caused by herpes zoster usually resolves after 1–2 weeks, the activated secondary autoimmune responses, such as myelitis, meningoencephalitis, acute cerebellar ataxia, and BBE, are much more serious (1–3). BBE has been considered an acute demyelinating disease of the brainstem caused by a direct infection by a pathogen or as a secondary autoimmune response, and characterized by a good prognosis to systemic treatment (4–6). Anti-sulfatide antibodies assume a pivotal role in the development of autoimmune-induced acute and chronic neuropathies (7). Our report has important implications for informing dermatologists of this neurological complication and advancing the diagnosis and treatment of this syndrome.

## Case presentation

An 83-year-old woman presented with neuralgic pain and the appearance of clusters of vesicles over the right shoulder and chest. She was diagnosed with herpes zoster and treated with intravenous valaciclovir every day for 2 weeks. Scabs gradually formed after treatment. However, progressive limb weakness, difficulty swallowing food, and disturbed consciousness occurred 4 weeks after the onset of herpes zoster.

Upon examination, the patient was somnolent. She presented with a Glasgow Coma Scale of 13 (E3V4M6). Her pupils were round, with the same diameter (3.0 mm), and pupillary response to light was preserved. However, the ocular movements, in all directions, were limited. Strength in all four limbs was grade 3. The muscle tone in all limbs was increased and the deep tendon reflexes were bilaterally increased. The Babinski sign was positive bilaterally. Unfortunately, the impairment of consciousness prevented the assessment of ataxia.

**Figure 1:**
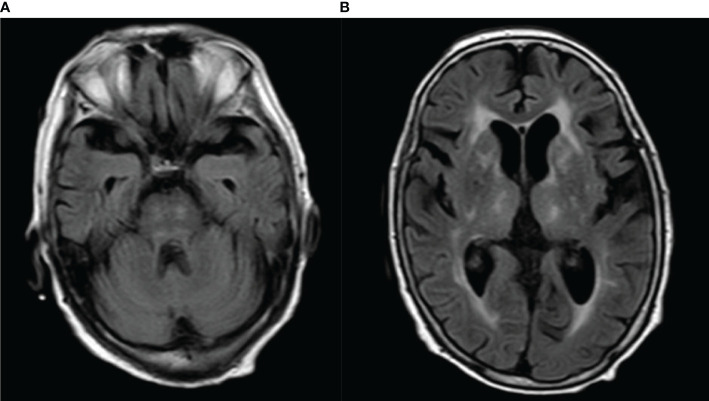
**(A, B)** FLAIR imaging revealed obvious high signal intensity in the pons and bilateral thalamus.

**Figure 2:**
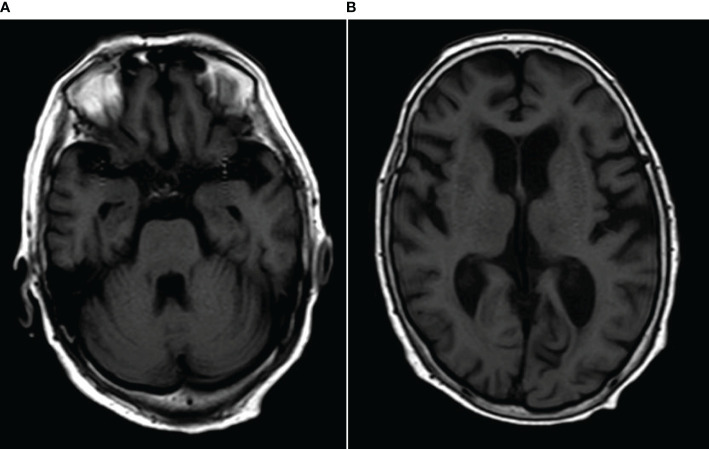
**(A, B)** T1-weighted images revealed low signal intensity in the pons and bilateral thalamus.

MRI showed abnormalities. FLAIR sequences revealed high signal intensity in the pons and bilateral thalamus suggestive of inflammation (**Figures 1A, B**). T1-weighted images revealed low signal intensity in the pons and bilateral thalamus (**Figures 2A, B**).

Cerebrospinal fluid (CSF) on day 2 after admission showed normal pressure and was clear with no pleocytosis. CSF revealed a white cell count of 4 cells × 10^6^/L, protein level of 329 (normal range: 200–400 mg/L), chloride level of 124 (normal range: 120–130 mg/L), and adenosine deaminase level of 0.4 (normal range: 0–8 U/L). CSF Gram staining, cryptococcal antigen, and acid-fast staining were negative. CSF cultures did not show any evidence of growth of bacteria, acid-fast bacillus, or fungi. Related autoimmune antibodies, including IgM and IgG antibody tests for sulfatide, GM1, GM2, GM3, GM4, GD1a, GD1b, GD2, GD3, GT1a, GT1b, and GQ1b (by enzyme-linked immunospot assay) were measured. The test results revealed a high anti-sulfatide IgM titer in the serum. We also used cell-based assays to measure IgG antibody for aquaporin-4, NMDAR1, AMPA1, AMPA2, LGI1, CASPR2, GABABR1, DPPX, lgLON5, GlyRα1, GABAARα1, GABAARβ3, mGluR5, D2R (DRD2), neurexin-3α, and GAD65, and results were all negative. Routine hematology and chemistry blood tests showed no evidence of metabolic disturbance, infection, or inflammation. The ultrasound of the abdomen and urinary system, computed tomography of the chest, and tumor marker investigations [including CEA, AFP, CA125 (glycoprotein), CA19-9, CA15-3, CA72-4, CYFRA 21-1, NSE, and squamous cell carcinoma antigen)], found no evidence of tumor.

Electroencephalography on day 4 revealed predominantly slow wave activity, suggesting an underlying encephalopathic process.

On day 4 after admission, the patient was given methylprednisolone intravenously, 80 mg/day for 5 days. After 5 days, the methylprednisolone dose was reduced to 40 mg/day. Oral methylprednisolone was tapered gradually and then stopped after 2 months.

By day 9 of her hospital stay, the patient’s consciousness level had significantly improved, and Glasgow Coma Scale was recorded as 15 (E4V5M6). Dysphagia and ophthalmoplegia had entirely resolved, and strength in all four limbs was grade 4. However, there was no significant change in muscle tone, deep tendon reflexes, or the Babinski sign.

By 2 weeks of her hospital stay, muscle tone in all limbs and the deep tendon reflexes were normal and the Babinski sign was negative bilaterally. No symptoms had recurred at her 3-month clinic follow-up.

## Discussion

BBE exhibits a central nervous system (CNS) predilection, reflected by altered consciousness, brainstem involvement, and long tract signs. Disordered consciousness to various degrees suggests that the brainstem reticular activating system is involved (8). MRI of the brain has been shown to be abnormal in only 11%–30% of patients with BBE, and it is useful to demonstrate brainstem lesions and distinguish BBE from Miller Fisher syndrome (MFS), neuromyelitis optica spectrum disorder (NMOSD), and other similar diseases (9). Many studies have shown that the usual abnormalities of MRI in BBE include T2 hyperintense signals in the brainstem (especially midbrain) and cerebellum (10).

In our case, the anti-sulfatide autoimmune antibodies were positive, and FLAIR imaging revealed obvious high signal intensity both in the pons and bilateral thalamus. In view of the acute bilateral ocular movement disorder, disturbance of consciousness, brisk deep tendon reflexes in upper and lower limbs, and bilateral positive Babinski sign, the probable diagnosis of Bickerstaff brainstem encephalitis was considered established.

When seeing a patient with brainstem encephalitis, the following differential diagnosis should be taken into consideration: listeria encephalitis, chronic lymphocytic inflammation with pontine perivascular enhancement responsive to steroids (CLIPPERS), and myelin oligodendrocyte glycoprotein antibody-associated disease (MOGAD) (11).

Infection should be considered first before diagnosing BBE. Potential infectious causes include listeria (12), enterovirus 71 (EV 71) (13), herpes viruses, and aspergillosis (14). Our patient had not been exposed to contamination from soft cheeses, unpasteurized milk, or deli meats. Results of her routine CSF parameters, like CSF white cell count, protein concentration, chloride level, and adenosine deaminase level, were considered normal. CSF Gram staining, cryptococcal antigen, and acid-fast staining were negative. CSF cultures did not show any evidence of growth of bacteria, acid-fast bacillus, or fungi. In addition, our patient’s condition improved markedly after treatment with methylprednisolone rather than with antimicrobial therapy.

Brainstem encephalitis is often confused with chronic lymphocytic inflammation with pontine perivascular enhancement responsive to steroids (CLIPPERS) (15). A comprehensive systematic review reported that ataxia (92.8%) was the most common symptom in CLIPPERS. Sixteen percent of the cases were associated with malignancy, mostly hematologic malignancies (16). Our ultrasound, computed tomography examination, and tumor marker investigations, found no evidence of a tumor. Along with typical responsiveness to steroids, patients with CLIPPERS have also been found to have high rates of clinical relapse following glucocorticoid tapering, requiring maintenance glucocorticoid or other immunosuppressive therapy (17). At her 3-month clinic follow-up, no symptoms had recurred in our patient. Despite the absence of gadolinium-injected sequences, we considered that BBE provided the most appropriate explanation for the clinical symptoms.

Myelin oligodendrocyte glycoprotein antibody-associated disease (MOGAD) is a group of central nervous system demyelinating diseases caused by autoantibodies against myelin oligosaccharide protein (MOG). MOGAD is typically associated with optic neuritis, transverse myelitis, or acute disseminated encephalomyelitis, and is less commonly associated with brainstem presentation, cerebral cortical encephalitis, or cerebellar presentations (18); and optic neuritis, particularly among adults, is the most common onset feature (19). Brainstem encephalitis is a kind of infrequent presentation in the clinical spectrum of MOGAD (20). In MOG-IgG-associated encephalomyelitis cases, patients with brainstem involvement account for about 30%, and isolated brainstem encephalitis that occurs without optic neuritis or myelitis is much rarer, accounting for only 1.8% (21). Brainstem encephalitis in MOGAD is characterized by diplopia, nystagmus, internuclear ophthalmoplegia, third nerve palsy, trigeminal hypesthesia, facial nerve paresis, dysarthria, and dysphagia (21). CSF pleocytosis, with white cell counts of >5 per µL, were found in over 50% of patients with a first demyelinating attack and MOG-IgG. More than 100 white blood cells per high-power field occurred in 12% of such patients (22). CSF protein was elevated in 30% of patients with a first demyelinating attack and MOG-IgG (23).

The clinical symptoms, MRI features, and CSF tests of our case were more characteristic of BBE rather than MOGAD. Unfortunately, the absence of MOG-IgG testing was a limitation of our report.

Although optimal effective treatment has not been established, most patients with BBE respond to intravenous immunoglobulin or methylprednisolone, either singly or in combination (24–26). A review of 27 articles on BBE, including 236 children, suggested that patients treated with any type of immunotherapy (steroid, intravenous immunoglobulins, or plasmapheresis) demonstrated faster resolution of symptoms than those receiving supportive care alone (27). A previous study suggested that sulfatide-positive patients, with either axonal neuropathy or a demyelinating condition, had a more severe disease compared to seronegative patients (28). However, methylprednisolone alone was effective, producing marked improvement, in our sulfatide-positive patient. Other authors have reported two cases of BBE showing a good therapeutic response to glucocorticoids alone (29, 30). We consider that secondary autoimmune responses activated by viral infection played an essential role in our patient, as shown by the very favorable response to systemic steroid therapy.

## Conclusions

BBE patients may present with symptoms of limb weakness, difficulty swallowing food, and impaired consciousness after herpes zoster, as in our case. This emphasizes the importance of a careful medical history, assessment of clinical symptoms, performing an MRI, testing autoimmune antibodies for rapid diagnosis, and ruling out differential diagnoses. Further studies, involving more patients with BBE with IgM anti-sulfatide autoantibodies, will contribute to a better understanding of the clinical characteristics and advance the diagnosis and treatment of this syndrome. Meanwhile, it is crucial for dermatologists to know about this severe neurological complication occurring after herpes zoster.

## Data availability statement

The original contributions presented in the study are included in the article/supplementary material. Further inquiries can be directed to the corresponding author.

## Ethics statement

Written informed consent was obtained from the individual(s) for the publication of any potentially identifiable images or data included in this article.

## Author contributions

XF: Writing – original draft, Writing – review & editing. QZ: Writing – original draft, Writing – review & editing. LZ: Writing – original draft, Writing – review & editing. XT: Writing – original draft, Writing – review & editing.
